# Epidemiological evaluation of concordance between initial diagnosis and central pathology review in a comprehensive and prospective series of sarcoma patients in the Rhone-Alpes region

**DOI:** 10.1186/1471-2407-10-150

**Published:** 2010-04-19

**Authors:** Antoine Lurkin, Francoise Ducimetière, Dominique Ranchère Vince, Anne-Valérie Decouvelaere, Dominic Cellier, François N Gilly, Dimitri Salameire, Pierre Biron, Guy de Laroche, Jean Yves Blay, Isabelle Ray-Coquard

**Affiliations:** 1 Centre Léon Bérard, 28 rue Laennec - 69008 Lyon; France; 2 INSERM EA 4129 « SIS », 28 rue Laennec - 69008 Lyon, France; 3 ONCORA network, Réseau Oncologie Rhône-Alpes BIOPARC/ADENINE - 60 Avenue Rockefeller, 69373 LYON Cedex 08, France; 4 CONCORDE network, Réseau Oncologie Rhône-Alpes BIOPARC/ADENINE - 60 Avenue Rockefeller, 69373 LYON Cedex 08, France; 5 ARC'ALPIN network, Unité de Concertation et de Recherche pour le Traitement des Affections Cancéreuses, CHU A. Michallon BP217 38043 GRENOBLE, France; 6 Institut de Cancerologie de la Loire, 108 Bis av. Albert Raimond 42270 Saint Priest en Jarez, France; 7 Merck Serono, 37 rue Saint Romain - 69008 Lyon, France; 8 INSERM U590 Cytokine et Cancer, 28 rue Laennec - 69008 Lyon, France

## Abstract

**Background:**

Sarcomas are rare malignant tumors. Accurate initial histological diagnosis is essential for adequate management. We prospectively assessed the medical management of all patients diagnosed with sarcoma in a European region over a one-year period to identify the quantity of first diagnosis compared to central expert review (CER).

**Methods:**

Histological data of all patients diagnosed with sarcoma in Rhone-Alpes between March 2005 and Feb 2006 were collected. Primary diagnoses were systematically compared with second opinion from regional and national experts.

**Results:**

Of 448 patients included, 366 (82%) matched the inclusion criteria and were analyzed. Of these, 199 (54%) had full concordance between primary diagnosis and second opinion (the first pathologist and the expert reached identical conclusions), 97 (27%) had partial concordance (identical diagnosis of conjonctive tumor but different grade or subtype), and 70 (19%) had complete discordance (different histological type or invalidation of the diagnosis of sarcoma). The major discrepancies were related to histological grade (n = 68, 19%), histological type (n = 39, 11%), subtype (n = 17, 5%), and grade plus subtype or grade plus histological type (n = 43, 12%).

**Conclusions:**

Over 45% of first histological diagnoses were modified at second reading, possibly resulting in different treatment decisions. Systematic second expert opinion improves the quality of diagnosis and possibly the management of patients.

## Background

Sarcomas are malignant tumors developing in soft tissue, bone, skin or internal organs. The large majority of soft tissue tumors are benign and 100 times more common than malignant lesions [1,2]. Because there are more than 50 histological subtypes of soft tissue sarcoma (STS) identified in the 2002 WHO classification, accurate diagnosis is difficult [3]. Given the rarity of the disease, inappropriate medical management has been reported in more than 70% of patients with sarcomas [4]. Careful pre-treatment evaluation is therefore essential for accurate diagnosis and appropriate treatment decision making [5,6]. Discrepancies between pathologists have been reported frequently [7-10]. Although immunohistochemistry, Fluorescence In Situ Hybridization (FISH) and molecular biology can facilitate diagnosis, these techniques are not routinely available in all laboratories and their use requires experienced pathologists with expertise in molecular biology.

Second opinion in diagnostic pathology has recently received considerable attention as a result of efforts to enhance institutional performance and reduce medical errors [11]. However, the mechanisms by which second opinion is obtained greatly influence the results [12]. Second opinions given by another institution or a specialty panel at the time of patient referral produce highly discordant rates as compared to analysis of cases referred to experts for review [13]. In the case of expert review, discrepancies are not viewed as "errors" but as a reflection of the acknowledged need for assistance. For this we initiated an exhaustive, prospective study involving the systematic comparison of initial histological diagnosis by a first ('non-expert') pathologist and second opinion (SO) from regional and/or national experts of the disease in a comprehensive population of patients diagnosed in a precise geographical region over a one-year period.

## Methods

### Objectives

The main objective of the present work was to evaluate the benefit of systematic central review by regional and national experts of all sarcoma cases diagnosed in the Rhône-Alpes region (RA). All sarcoma cases diagnosed by pathologists of the region were reported, and data were statistically analyzed to quantify inter-observer differences and determine their nature.

### Description of the Region

RA is the second largest region in France, with nearly 6 million inhabitants and 8 departements (i.e. the administrative and geographical unit in France). It has 15 public structures (3 university hospitals, 1 cancer center, 11 general hospitals) with 59 pathologists and 28 private structures with 80 pathologists.

### Patient selection

To be eligible, patients had to meet the following inclusion criteria: first diagnosis of connective tissue tumor, according to the 2002 WHO definition, and no previous treatment, first sarcoma diagnosis between March 1, 2005 and February 28, 2006 in RA region, all disease stages, and age ≥ 15 years. Exclusion criteria were as follows: diagnosis of relapse or a diagnosis other than sarcoma (i.e. low grade phyllodes tumor). Similarly, patients were not included when primary diagnosis had been established in the reference center (Leon Berard Cancer Center) by one of the 'experts' or if the tumor specimen was not sent to the expert or when there was not enough tumor material. In order to include patients and assess the concordance our study obtained an ethical approval by a review board (CNIL: Commission Nationale de l'information et des libertés. Independent administrative authority protecting privacy and personal data). The evaluation of medical records has been made through clinical audit

### Study design

The goal of this study was to compare initial histological evaluation by the diagnostic pathologist (generally not an expert on these diseases) and results of the central expert review (CER) by two regional and national exclusively soft tissue experts. These two experts, members of the French Sarcoma Group (FSG), were selected by the EMS project scientific committee. All pathologists working in the region agreed to cooperate. All suspected cases of sarcoma (soft tissue, bone and visceral tissue sarcoma; n = 671) diagnosed during the reference period were collected [14].

For each patient, a copy of the original histology report and histopathological specimens (Hemalin Eosin Safran (HES) and paraffin-embedded tissue) representative of the tumor sample were provided.

The diagnostic pathologist was offered financial compensation for each patient included (50 euros). For all included patients, immunohistochemistry (and/or molecular biology analysis) was performed by the referent pathologist.

### Exhaustiveness control

To ensure exhaustiveness, several controls were established throughout the study by comparing the registered patients with the complete list of: 1) sarcomas reviewed by the expert pathologist every two months during the inclusion period; 2) medical files of the *multidisciplinary sarcoma committee and *patients obtained from the Medical Information Department of the reference center; 3) pediatric patients with sarcoma to confirm the number of patients aged 15-18 and obtained from the pediatric registry of the RA region; 4) all sarcomas diagnosed by initial pathologists; then we excluded patients with exclusion criteria. After cross validation, only 5 additional patients were identified as missing and included [15,16].

### Main outcome measure

Differences between the first diagnosis established by the non-expert pathologist and the second opinion given by experts were evaluated and scored on a three-point scale: The two experts evaluated in same time the diagnosis.

Zero agreement corresponded to cases where initial diagnosis was benign and final diagnosis was malignant (sarcoma) or conversely, or where the tumor was classified in different histological subtypes (i.e. synovialosarcoma vs. liposarcoma).

Partial agreement corresponded to cases where both pathologists diagnosed a sarcoma but with different histopathological grades, or with a different subtype (i.e. dedifferentiated liposarcoma vs. myxoid round cell liposarcoma).

Full agreement corresponded to cases where both observers gave identical diagnoses.

To define this score, the two experts must conclude to the same diagnose. In some rare cases and when the two experts did not have the same conclusion or if the diagnose was difficult the diagnosis was reexamined either by another expert (international expert Pr Fletcher) or/and discussed at monthly FSG pathologist meetings and a final consensus was determinated.

For all sarcoma types with mutation, a molecular biology was systematically assessed (FISH technic, PCR or DNA sequencing) to characterize the genetic alteration and confirm the diagnose. The Immunomarques were systematically done again by the expert

### Grading system

The grading system of the French Sarcoma Group of the French Federation of Cancer Centers (FNCLCC) was used in this study as now proposed in the WHO classification [17]. Grade was rated 'not applicable' for some specific histological types (e.g. Kaposi sarcoma) or when the grade could not be determined (biopsy specimen).

### Subgroup analysis

For all included cases, the pathologist investigators were systematically offered an expert second opinion and the discrepancies were analyzed. However, two groups of patients were distinguished. The first one ("requested SO" group) corresponded to patients examined by a "non expert" pathologist who requested a second opinion from experts to confirm initial diagnosis. The second group ("control" group) included patients whose tumor samples were analyzed by a 'non expert' pathologist who did not request confirmation of diagnosis by experts and whose findings were reviewed only in the context of the present project but the results were not disclosed.

### Statistical analysis

To evaluate patient characteristics and diagnostic concordance, categorical data were analyzed using Pearson's χ^2^-test or Fisher's exact test, as appropriate. Continuous data were analyzed with Student's *t*-test. The statistical significance level was set at *p *= 0.05 in a two-sided test.

Comparisons were also made between the "requested SO" group and controls. The χ^2 ^test was used to determine the rate of concordance and the types of discordance. Correlations between the most frequent causes of error and groups were analyzed using the Kappa test. For grading evaluation, a two by two table was constructed to compare original diagnosis against final diagnosis, both subsets being partitioned according to whether or not the diagnosis was the type of sarcoma under consideration. A Kappa test was used to measure agreement between initial diagnosis and expert review as compared to what would be expected by chance alone [18,19]. All analyses were performed using SPSS^® ^(version 12.0) and SAS^® ^softwares.

## Results

All values reported hereafter for grade, histology and type or site of sarcoma are those obtained after expert review.

### Characteristics of selected patients and tumors

**Figure 1:**
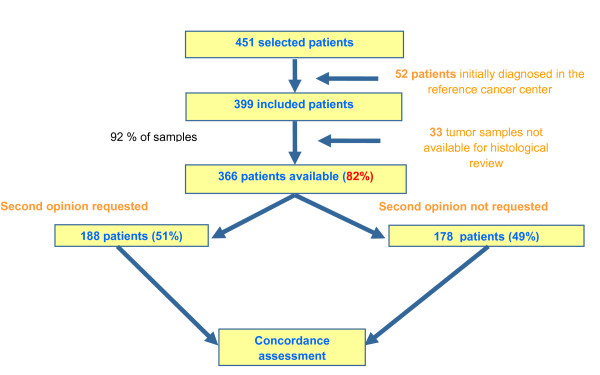
**Patient selection**.

Of 671 patients initially screened by pathology laboratories, 220 (33%) were excluded, either because of patient age < 15 (n = 26, 4%), local relapse (n = 92, 14%), metastatic relapse (n = 40, 6%), or because the patient had not been firstly diagnosed in RA (n = 36, 5%) or had not been diagnosed between March 2005 and Feb 2006 (n = 26, 4%). Among the 451 selected patients, 52 (12%) were further excluded because initial diagnosis had been established in the reference center by one of the experts. Of the 399 remaining patients, 29 (7%) were excluded because there was no or not enough tumor tissue available for a second histological analysis and 4 (1%) because the initial histological report was not available. Finally, 366 (92%) patients were eligible for final analysis (Figure 1). The first diagnosis of sarcoma was performed in private practice for 265 (72%) patients and in public laboratories for 101 (28%) patients. Only 2 laboratories did not recruit any patient.

### Characteristics of included patients and tumors

Patients' characteristics are reported in Table 1. Among the 366 analyzed patients, 184 were males (50%) and 182 were females (50%). Median age was 61 years (range, 15-92): 60 (range, 15-92) for males and 63 (range, 16-91) for females.

**Table 1:** Characteristics of included patients

	Number	%
Included patients	366	100%

***Types of sarcoma***		
Soft tissue	215	59
Visceral tissue	130	35
Bone tissue	21	6

***Tumor site***		
Abdomen	101	27.6
Lower limb	76	20.8
Thorax	54	14.8
Pelvis	45	12.3
Upper limb	34	9.3
Head and Neck	29	7.9
Retroperitoneum	21	5.7
Multiple localizations	5	1.4
Axial skeleton	1	0.3

***Histological subtype***		
GIST	65	17.8
Liposarcoma	56	15.3
NOS Sarcoma *	52	14.2
Other **	38	10.3
Leiomyosarcoma	26	7.1
Dermatofibrosarcoma protuberans	20	5.5
Kaposi sarcoma	18	4.9
Uterine leiomyosarcoma	12	3.3
Myxofibrosarcoma	12	3.3
Osteosarcoma	11	3.0
Angiosarcoma	10	2.7
Chondrosarcoma	9	2.5
PNET/Ewing sarcoma	8	2,2
Rhabdomyosarcoma	8	2.2
Synovial sarcoma	7	1,9
Unclassified malignant connective tumors	14	3.8

***Grade***		
I	85	23.2
II	77	21.0
III	97	26.5
Not applicable***	103	28.1
Unknown	4	1.1

* NOS = not otherwise specified.

**The 'other' category included sarcoma subtypes with less than 5 patients and tumors initially described as sarcomas and reclassified by the experts as carcinomas or sarcomatoid carcinomas

**Not applicable (Kaposi sarcoma, for instance)

### Concordance analysis

Concordance analysis was performed on 93 (25%) sarcoma biopsy specimens and 273 (75%) surgical samples. Concordance data regarding grade, type of tumor sample (biopsy vs. surgery) and type of laboratory (private vs. public) are given in Table 2. Concordance analysis showed 70 cases of zero agreement (19%), 97 partial agreements (27%) and 199 full agreements (54%). The rate of discordance was higher in grade II-III than in grade I tumors (*p = 0.012)*. On the other hand, no significant differences were reported according to type of tumor sample, type of laboratory and molecular biology examination (yes vs. no) (Table 2). The molecular biology techniques used were the Fluorescence in situ hybridization (FISH), the PCR and DNA sequencing.

**Table 2:** Concordance analysis

*Concordance*	Zero	Partial	Full	*p*
Included tumors	70 (100%)	97 (100%)	199 (100%)	
***Type of laboratory***				
**Public**	17 (16.8%)	28 (27.7%)	56 (55.4%)	***0.78***
**Private**	53 (20%)	69 (26.%)	143 (54%)	

*Concordance*	**Zero**	**Partial**	**Full**	***p***

Included tumors	70	97	97	
***Type of tumor sample***				
**Biopsy**	16 (17.2%)	23 (24.7%)	54 (58.1%)	***0.70***
**Surgical specimen**	54 (19.8%)	74 (27.1%)	145 (53.1%)	

*Concordance*	**Zero**	**Partial**	**Full**	***p***

Included tumors	44	81	134	
***Grade***				
**I**	9 (10.6%)	21 (24.7%)	55 (64.7%)	***0.01***
**II-III**	35 (20.1%)	60 (34.5%)	79 (45.4%)	

*Concordance*	**Zero**	**Partial**	**Full**	***p***

Included tumors	67	97	199	
***Molecular biology study***				
**No**	45 (20%)	61 (26.%)	127 (54%)	***0.84***
**Yes**	23(17.6%)	36 (27.5%)	72 (55.0%)	

Causes of non-concordance are presented in Table 3. Discrepancies between initial diagnosis and review were essentially related to grade and histological subtype. Details of zero and partial agreements are given in Table 4 and Additional File 1, respectively. More precisely, in the zero concordance group, the diagnoses were changed from benign to malignant for 7 patients. The modification of diagnosis between malignant tumor and benign have been concerning 3 patients. The most frequent discrepancy was related to the grade of the tumor: either no grading by the diagnostic pathologist while the expert attributed grade 3 (n = 33, 20%), or misinterpretation of the grading with grade 3 attributed by the diagnostic pathologist and grade 1 by the expert (n = 3, 2%). Other (n = 131, 78%) discrepancies were related to the grade (0 vs. I, I vs. II, II vs. III) and histological subtype. We measured the chance-corrected agreement on grade between groups using the Kappa test. The Kappa coefficient was 0.7857 (p < 0.0001), which reflected a high level of agreement. The major cause of discrepancies was the very high proportion (n = 85, 66%) of tumors not graded by diagnostic pathologists. However, when diagnostic pathologists did grade the tumors, their grading was generally correct (Additional File 1).

**Table 3:** Reasons for non-concordance

Reasons for non-concordance	Frequency	%
Grade	68	18.5
Histological type	39	10.7
Grade and Histological type	30	8.2
Subtype	17	4.6
Grade and subtype	13	3.6
		
**SUB-TOTAL**	**167**	**45.6**
*Patient with total agreement*	*199*	*54.4*
***TOTAL***	366	*100*

**Table 4:** Diagnostic differences in cases with zero concordance

Expert diagnosis	Initial diagnosis	Frequency (n = 70)
NOS Sarcoma	MPNST	1
NOS Sarcoma	Angiosarcoma	1
NOS Sarcoma	Unclassified malignant tumor	6
NOS Sarcoma	Leiomyosarcoma	1
NOS Sarcoma	GIST	1
NOS Sarcoma	Chondrosarcoma	1
NOS Sarcoma	Embryonic Rhabdomyosarcoma	1
NOS Sarcoma	Benign Tumor	1
NOS Sarcoma	Liposarcoma	1
NOS Sarcoma	Leiomyosarcoma	2
Leiomyosarcoma	Benign Tumor	2
Leiomyosarcoma	NOS Sarcoma	1
Osteosarcoma	NOS Sarcoma	1
Osteosarcoma	Unclassified malignant tumor	1
Osteosarcoma	Melanoma	1
Lipoma-like Liposarcoma	Benign Tumor	1
Lipoma-like Liposarcoma	Fibrosarcoma	1
Lipoma-like Liposarcoma	Unclassified malignant tumor	1
Liposarcoma	Benign Tumor	1
Liposarcoma	NOS Sarcoma	2
Dedif. Liposarcoma	Leiomyosarcoma	2
Dedif. Liposarcoma	Unclassified malignant tumor	1
Dedif. Liposarcoma	NOS Sarcoma	1
Myxofibrosarcoma	Liposarcoma	1
Myxofibrosarcoma	NOS Sarcoma	3
Myxofibrosarcoma	Sarcomatoid Carcinoma	1
Myxofibrosarcoma	Chondrosarcoma	1
Sarcomatoid Carcinoma	Leiomyosarcoma	1
Sarcomatoid Carcinoma	NOS Sarcoma	3
Angiosarcoma	Unclassified malignant tumor	1
Angiosarcoma	Synovial sarcoma	1
Benign Tumor	Inflammatory MyxofibroSarcoma	1
Benign Tumor	Leiomyosarcoma	1
Benign Tumor	Well-differenciated lipoma-like Liposarcoma	1
Epithelioid Sarcoma	Unclassified malignant tumor	1
Epithelioid Sarcoma	Synovial sarcoma	1
PNET/Ewing sarcoma	Carcinoma	2
PNET/Ewing sarcoma	Unclassified malignant tumor	3
Rhabdomyosarcoma	Leiomyosarcoma	1
GIST	NOS Sarcoma	1
Carcinoma	Phyllodes Tumor	1
Carcinoma	NOS Sarcoma	1
Solitary fibrosis/malignant Tumor	GIST	1
Uterine Leiomyosarcoma	Benign Tumor	1
PEComa	Unclassified malignant tumor	1
Fibrosarcoma	Unclassified malignant tumor	1
Endometrial Stromal Sarcoma	Leiomyosarcoma	1
Epithelioid Hemangioendothelioma	Carcinoma	1
Inflammatory Myxofibrosarcoma	Epithelioid Sarcoma	1
Unclassified malignant tumor	Carcinoma	1
Low grade fibromyxoid Sarcoma	Benign Tumor	1
Kaposi sarcoma	NOS Sarcoma	1
Synovial sarcoma	Benign Tumor	1
Dermatofibrosarcoma protuberans	Benign Tumor	1

NOS: not otherwise specified

PNET: primitive neuroectodermal tumor

GIST: gastrointestinal stromal tumor

PEComa: malignant perivascular epithelioid cell tumor

MPNST: malignant peripheral nerve sheath tumor

The second most frequent discrepancy was related to the histological type (Table 4). In 22 (31%) patients the diagnosis reviewed and modified by the expert concerned benign tumor or sarcomatoid carcinoma.

Diagnosis was confirmed by molecular biology (RT-PCR, FISH) in 131 (36%) patients: 72 (55%) in the "requested SO" group and 59 (45%) in the control group at the request of the expert reviewer. Seventy-two (55%) tests were performed in the group with full concordance, 36 (27%) with partial concordance and 23 (18%) with zero concordance. Eighty-seven (66%) of these 131 molecular biology tests were positive, 32 (24%) were negative and 12 (9%) were non significant. They were performed essentially in patients with GIST (n = 41, 31%), liposarcoma (n = 39; 30%) and dermatofibrosarcoma protuberans (n = 11, 8%). Final diagnosis could not be established by the first expert for 20 (5%) patients, and the case was submitted to other sarcoma experts for second opinion (French sarcoma group).

### Requested SO group versus control group

For the two groups of patients distinguished, the characteristics are compared in Tables 5 and Additional File 2.

**Table 5:** Patient characteristics per subgroup

Patient characteristics.			
	**requested SO group**	**Control group**	***p***

Included patients	188 (100%)	178 (100%)	

*Sex*			
**Males**	102 (54.3%)	82 (46.1%)	*0.117*
**Females**	86 (45.7%)	96 (53.9%)	

*Age (years)*			
**Mean**	57.9	59.6	
**Median**	62	61	*0.359*
**Range**	[15-86]	[18-92]	

*Types of tumor*			
**Soft tissue**	117 (62.2%)	98 (55.1%)	
**Bone tissue**	13 (6.9%)	8 (4.5%)	*0.128*
**Visceral tissue**	58 (30.9%)	72 (40.4%)	

*Localization*			
**Upper limb**	15 (8.%)	19 (10.7%)	
**Lower limb**	45 (23.9%)	31 (17.4%)	
**Abdomen**	44 (23.4%)	57 (32%)	
**Thorax**	31 (16.5%)	23 (12.9%)	*0.313*
**Head and Neck**	16 (8.5%)	13 (7.3%)	
**Pelvis**	25 (13.3%)	20 (11.2%)	
**Retroperitoneum**	10 (5.3%)	11 (6.2%)	
**Axial skeleton**	1 (0.5%)	0 (0%)	
**Multiple localizations**	1 (0.5%)	4 (2.2%)	

*Grade*			
**Grade I**	47 (25%)	38 (21.3%)	
**Grade II**	36 (19.1%)	41 (23%)	
**Grade III**	50 (26.6%)	47 (26.4%)	*0.716*
**Non applicable**	52 (27.2%)	51 (28.7%)	
**Unknown**	3 (1.6%)	1 (0.6%)	

*Histological subtypes*			
**Liposarcoma**	37 (19.7%)	19 (10.7%)	
**NOS Sarcoma**	32 (17.0%)	20 (11.2%)	
**GIST**	23 (12.2%)	42 (23.6%)	
**Myxofibrosarcoma**	9 (4.8%)	3 (1.7%)	
**Leiomyosarcoma**	7 (3.7%)	19 (10.7%)	
**PNET/Ewing**	6 (3.2%)	2 (1.1%)	
**Rhabdomyosarcoma**	6 (3.2%)	2 (1.1%)	* < 0.001.*
**Chondrosarcoma**	6 (3.2%)	3 (1.7%)	
**Dermatofibrosarcoma**	4 (2.1%)	16 (9.0%)	
**protuberans**			
**Uterine Leiomyosarcoma**	5 (2.7%)	7 (3.9%)	
**Osteosarcoma**	5 (2.7%)	6 (3.4%)	
**Kaposi Sarcoma**	5 (2.7%)	13 (7.3%)	
**Synovialosarcoma**	5 (2.7%)	2 (1.1%)	
**Angiosarcoma**	3 (1.6%)	7 (3.9%)	
**Unclassified malignant tumor**	7 (3.7%)	7 (3.9%)	
**Other**	28 (14.9%)	10 (5.6%)	

Only three items seemed different between subgroups: type of samples, type of laboratory and histological subtype. The patients for whom initial diagnosis had been made from biopsy specimens were most frequently not proposed for a SO. Likewise, the majority of patients included in the group without SO were first diagnosed in public laboratories. For all other factors no differences were noted. In fact, non expert pathologists asked for a second expert opinion principally for difficult diagnoses or cases requiring molecular analysis (n = 188). Sex, age, type of tumor, or tumor localization and grade did not influence the decision of the first pathologist to request a second opinion (Table 5).

### Concordance results in the two subgroups of patients

The concordance observed between the two groups of patients is described in Table 6. Not surprisingly, concordance was significantly better in cases where the primary pathologist did not ask for a second opinion as compared to cases where an expert second opinion was requested spontaneously (66% vs. 44%) (*p < 0.001*). Reasons for non-concordance were tested in both groups. The type of sample and the type of laboratory were not found correlated to concordance, with respectively 40 (70%) vs. 77 (63%) (*p = 0.62*), and 82 (68%) vs. 35 (60%) (*p = 0.39*) full concordance in the group without SO. In the group with SO, full concordance results were 14 (39%) vs. 68 (44%) (p = 0.72) and 61 (42%) vs. 21 (48%) (*p = 0.66*).

**Table 6:** Comparison of concordance results in the two groups

	Requested SO group	Control group	*p*
Included patients	188 (100%)	178 (100%)	

*Concordance*			
**Zero**	53 (28.2%)	17 (9.6%)	
**Partial**	53 (28.2%)	44 (24.7%)	* < 0.001.*
**Full**	82 (43.6%)	117 (65.7%)	

Included patients	106 (100%)	61 (100%)	

*Type of discordance*			
**Subtype alone**	10 (9.4%)	7 (11.5%)	
**Grade alone**	31 (29.2%)	37 (60.7%)	
**Histological type alone**	27 (25.5%)	12 (19.7%)	* < 0.001*
**Grade + Subtype**	13 (12.3%)	0 (0%)	
**Grade + Histological type**	25 (23.6%)	5 (8.2%)	

## Discussion

Full concordance was reported for only 54% of cases included in this comprehensive cohort of sarcoma patients treated in RA in one year. Indeed, accurate diagnosis is essential to ensure appropriate management of patients with sarcoma, especially in the context of new targeted therapies. This study confirmed that centralized pathological review improves the quality of diagnosis in these rare tumors.

This study confirms that the diagnosis of sarcoma is very difficult to establish since more than 45% of first diagnoses were declared invalid by the panel of experts conducting the centralized pathological review. The major result was that concordance seems independent of the type of laboratory providing the primary diagnosis, the nature of the tumor samples or the tissue affected by the sarcoma (bone, soft tissue, viscera). The most frequent discrepancies identified were related to tumor grade and histological type. Exact determination of the tumor type and grade is crucial for making individual treatment decisions and subsequently improving patient outcome [20,21]. In fact, determining the grade of the tumor is essential to decide between adjuvant chemotherapy or not, neo-adjuvant chemotherapy or not, or radiotherapy or not. With the introduction of targeted treatments and the proliferation of clinical studies, patients whose grade has not been correctly evaluated may be excluded from trials [20-23]. Although the clinical practice recommendations published in France have confirmed that the tumor grade must be included in the histological report generated at the time of diagnosis, this information is not always given, as confirmed in other studies demonstrating that the reproducibility of grade is very difficult to achieve [20,23-25]. On the other hand, when non expert pathologists participating in the study did grade a tumor, their evaluation was generally correct. Pathologists may sometimes lack experience and would benefit from training sessions organized in the framework of continuous medical education [26].

Accurate determination of the type of sarcoma lesion is also crucial for correct patient management especially for differentiating between benign and malignant tumors, or between the different subtypes [27]. Determining the grade of the tumor is more reproducible than characterizing its histological type which is the second cause of diagnostic discordance [8]. The second cause of discrepancies concerned the histological type, and the central question that arises is whether all sarcoma cases should be reviewed in a specialized center. Importantly, all pathologists evaluating sarcoma patients should be able to use immunohistochemistry for confirmation of diagnosis. For certain histological types (GIST, Dermatofibrosarcoma protuberans...), molecular biology can also contribute to the establishment and/or the confirmation of diagnosis [28-32]. Non concordance between first diagnosis and review seems very frequent for liposarcoma, PNET tumor and NOS sarcoma, and it is high for GIST and dermatofibrosarcoma protuberans for which specific markers are available. Similar results were reported by Harris et al, with a high degree of agreement for osteosarcoma and chondrosarcoma and low agreement for leiomyosarcoma and malignant fibrous histiocytoma [27,28].

In the literature, the rate of diagnostic errors in patients with soft tissue sarcoma is between 25% and 40%. [6-10,33-37], Surprisingly, since the first published reports on second opinion for sarcoma tumors in 1980, despite the introduction of new tools (immunochemistry, molecular biology etc.) and the development of educational workshops, the percentage of concordance has remained unchanged. Disagreements between diagnostic pathologists and expert panel members are inescapable (unrepresentative samples, heterogeneous tumors, misdiagnosis of grading...). In spite of the relatively high incidence of sarcoma, such variations in diagnosis will continue to occur and no significant convergence can be expected over time [14]. Our comprehensive prospective study confirmed the results of previous retrospective studies, with inconsistencies between primary diagnosis and histological review in 45% of all cases. Our results revealed that diagnostic pathologists were often lack expertise in this disease or in other rare tumors. Thus, systematic expert second opinion seems essential [35]. The inexperience of non-specialists with the multitude and complexity of soft tissue sarcomas is probably the most important factor accounting for diagnostic discrepancies [27]. Actually, this question of centralized diagnosis is less relevant for tumors with more "standardized" diagnosis and management, like carcinoma or ovarian cancer [38,39].

In this study, 50% of the tumors were spontaneously addressed for a second opinion by the non-expert pathologist, versus only 40% in a previous study[25]. All pathologists in the RA region took part in the study and full comprehensiveness was achieved. However, to achieve such a successful recruitment, many requirements must be met. In particular, there must be a key opinion leader and expert pathologist with recognized expertise in the field. Financial support to the pathologists participating in the study also seems essential. Finally, it is important, to keep the pathologists informed through regular meetings and newsletters. They must be involved and get feedback about the final diagnosis of their patient as well as about the advancement of the study. Cancer network participation can facilitate the involvement of pathologists and ensure exhaustiveness. It is well established in sarcoma pathology that "expert" opinion is not always absolutely convergent, though often different conceptualizations of a tumor do not necessarily generate different treatment implications. With the help of CONTICANET (CONnective TIssue CAncer NETwork), a similar prospective study has been initiated in the Aquitaine (France) and Venetia (Italy) regions to compare the rate of diagnostic discordance and confirm our conclusions. The correlation between concordance and free survival will be essentially evaluated on grade II/III tumors. The assessment of concordance is important for the patient for its impact on the diagnosis. The optimal diagnosis but also the optimum treatments (R0, radiotherapy decision) have an important role. Their prognostic value will be determinate in another article.

## Conclusion

In conclusion, the inexperience of non-specialized pathologists with the multitude and complexity of sarcoma tumors, and the non availability of new molecular diagnostic tools are the most important factors accounting for diagnosis discrepancies. A centralized pathological review, a rapid and efficient help with access to molecular biology analysis seem of vital importance in these rare tumors. More efficient information and education of the pathologists also seems essential to ensure accurate diagnosis and grading [31].

## Competing interests

I declare that all authors disclose any financial and personal relationships with other people or organisations that could inappropriately influence (bias) their work. We would like just specify that Dr Dominic Cellier is the scientific relationship director of Merck-Serono. Merck-Serono is our financial support.

## Authors' contributions

AL: Data collection, data analyse, interpretation data, statistical analyse, article writing; FD: Data collection, data analyse, interpretation data, written participation; DR-V: Data collection, histological review, interpretation data, final article review; A-VD: Data collection, histological review, interpretation data, final article review; DC: Study design, administrative participation, final article review; FG: Data collection, administrative participation, final article review; DS: Data collection, data analyse, final article review; PB: Data collection, administrative participation, final article review; GDL: Data collection, administrative participation, final article review; J-YB: Project conception, study design, data collection, final article review; IR-C: Project conception, study design, data collection, statistical analyse, final article review. All authors read and approved the final manuscript

## Pre-publication history

The pre-publication history for this paper can be accessed here:

http://www.biomedcentral.com/1471-2407/10/150/prepub

## Supplementary Material

Additional file 1 Table S1: Grade concordance between groups using the Kappa test (n = 157). Click here for file

Additional file 2 Table S2: Characteristics of patients per type of laboratory and type of tumor sample. Click here for file
