# Supplementary material for: Epidemiological evaluation of concordance between initial diagnosis and central pathology review in a comprehensive and prospective series of sarcoma patients in the Rhone-Alpes region
Source: BMC Cancer. 2010 Apr 19;10:150. doi: 10.1186/1471-2407-10-150 (PMC2873387; doi:10.1186/1471-2407-10-150)
Supplement: Additional file 1 — Table S1: Grade concordance between groups using the Kappa test (n = 157). [file 1471-2407-10-150-S1.DOC]

Additional files

Additional file 1, Table S1: Grade concordance between groups using the Kappa test (n=157)

| **Initial decision**  **Expert decision** | Grade 1  (N = 63) | Grade 2  (N = 42) | Grade 3  (N = 52) |
| --- | --- | --- | --- |
| Grade 1 | 53 (84.1) | 2 (4.8) | 3 (5.8) |
| Grade 2 | 9 (14.3) | 32 (76.2) | 4 (7.7) |
| Grade 3 | 1 (1.6) | 8 (19.1) | 45 (86.5) |
