# Supplementary material for: Epidemiological evaluation of concordance between initial diagnosis and central pathology review in a comprehensive and prospective series of sarcoma patients in the Rhone-Alpes region
Source: BMC Cancer. 2010 Apr 19;10:150. doi: 10.1186/1471-2407-10-150 (PMC2873387; doi:10.1186/1471-2407-10-150)
Supplement: Additional file 2 — Table S2: Characteristics of patients per type of laboratory and type of tumor sample. [file 1471-2407-10-150-S2.DOC]

Additional file 2, Table S2: Characteristics of patients per type of laboratory and type of tumor sample

| **Characteristics of patients.** | | | |
| --- | --- | --- | --- |
|  | Requested SO group | Control group | ***p*** |
| Included patients | 188 (100%) | 178 (100%) |  |
| *Type of laboratory**  **Public**  **Private** | 43 (42.6%)  145 (54.7%) | 58 (57.4%)  120 (55.8 %) | ***0.038*** |
| *Type of sample **  **Biopsy**  **Surgical specimen** | 36 (38.7%)  152 (55.7%) | 57 (61.3%)  121 (44.3%) | ***0.005*** |
